# Impact of molecular sequence data completeness on HIV cluster detection and a network science approach to enhance detection

**DOI:** 10.1038/s41598-022-21924-8

**Published:** 2022-11-10

**Authors:** Sepideh Mazrouee, Camden J. Hallmark, Ricardo Mora, Natascha Del Vecchio, Rocio Carrasco Hernandez, Michelle Carr, Marlene McNeese, Kayo Fujimoto, Joel O. Wertheim

**Affiliations:** 1 Department of Medicine, University of California San Diego, San Diego, CA USA; 2 Houston Health Department, Houston, TX USA; 3 Department of Medicine, University of Chicago, Chicago, IL USA; 4 Instituto Nacional de Enfermedades Respiratorias “Ismael Cosío Villegas”, Mexico City, México; 5 Department of Health Promotion and Behavioral Sciences, The University of Texas Health Science Center at Houston, Houston, TX USA

**Keywords:** Epidemiology, Computational models

## Abstract

Detection of viral transmission clusters using molecular epidemiology is critical to the response pillar of the Ending the HIV Epidemic initiative. Here, we studied whether inference with an incomplete dataset would influence the accuracy of the reconstructed molecular transmission network. We analyzed viral sequence data available from ~ 13,000 individuals with diagnosed HIV (2012–2019) from Houston Health Department surveillance data with 53% completeness (n = 6852 individuals with sequences). We extracted random subsamples and compared the resulting reconstructed networks versus the full-size network. Increasing simulated completeness was associated with an increase in the number of detected clusters. We also subsampled based on the network node influence in the transmission of the virus where we measured Expected Force (ExF) for each node in the network. We simulated the removal of nodes with the highest and then lowest ExF from the full dataset and discovered that 4.7% and 60% of priority clusters were detected respectively. These results highlight the non-uniform impact of capturing high influence nodes in identifying transmission clusters. Although increasing sequence reporting completeness is the way to fully detect HIV transmission patterns, reaching high completeness has remained challenging in the real world. Hence, we suggest taking a network science approach to enhance performance of molecular cluster detection, augmented by node influence information.

## Introduction

The ability to localize and monitor high prevalence regions and disproportionately affected populations in transmission networks by using molecular data has become central to guiding HIV prevention interventions. In the United States, clinical HIV data reported to public health surveillance are used for epidemiology and prevention. In 2018, CDC recommended that all jurisdictions use HIV genetic sequence data from clinical drug-resistance tests to identify people living with HIV in “clusters” of others with genetically similar strains. Phylogenetic analysis of such molecular data to determine genetic relatedness is computationally intensive and makes continuous state or national level analysis of HIV transmission networks labor-intensive. Consequently, many practical approaches employ graph theory and track relatedness among groups of similar viral genomes implying epidemiological connections^1–3^. These methods connect similar HIV-1 *pol* sequences in a graph to form clusters of transmission. Detection of molecular clusters became increasingly expeditious by using HIV sequence data from clinical drug-resistance tests and an analysis tool, Secure HIV-TRACE, to identify groups of people with genetically similar strains. Identifying growing clusters that represent rapid transmission, allows public health officials to tailor prevention interventions^4–6^. Review of intervention outcomes during response efforts to clusters has shown notable benefits in prevention and care service uptake and evidence of reduced transmission^7^. However, HIV cluster response is inextricably tied to the methodology of detection based on surveillance data. Apart from the existence of noise, inconsistent data format, and other challenges in real-world data, we often face data missingness or incompleteness in health applications^8–11^. Data incompleteness or imperfection, such as unreported data (known as partial missingness), can imply data collection methodological errors. In the case of health department surveillance records, this may also reflect undiagnosed asymptomatic positive cases which is a known factor in transmittable diseases. If missingness happens frequently and no information is provided for one or more variables or for an entire person, the implications of the missing data (undiagnosed or out-of-care people living with HIV) might be inevitable. By definition, data completeness denotes the degree to which all relevant data are available in the dataset. Understanding the reasons why data are missing is important for handling the remaining data correctly^12,13^.

In the U.S., it is estimated that 1.2 million people are living with HIV among^14^ of whom nearly 28,000 live in Houston/Harris County^15^. In the U.S., HIV molecular networks are reconstructed by performing retrospective analyses utilizing drug resistance data to understand the dynamics of HIV clusters or outbreaks^16–20^. Once dynamics are understood, health departments can determine opportunities for HIV prevention and care and, subsequently, improve service delivery. However, such sequence data are collected only from HIV diagnosed and in-care populations, leaving many persons with HIV (PWH) who have no access to consistent care out of cluster detection and response. Therefore, the gap in access to the full dataset might affect our understanding of the dynamics of transmission^21^. In fact, the level of molecular data completeness varies not only geographically between states but also fluctuates across years. For instance, Michigan and Washington were reported nationally as having the highest completeness of reported HIV sequence data (in 2015) at 73% and 66% respectively^21^. At the time of this study, we imagine the occurrence of the COVID-19 pandemic in 2020 had a negative impact on sequence data collection for PWH that requires investigation. We analyzed the data from 12,818 newly diagnosed PWH reported to the Houston Health Department (HHD) between 2012 and 2019. The genotype data completeness rate was estimated at approximately 50% of PWH. The intention of this study is to quantify how the incompleteness of HIV molecular data can affect the reconstruction of HIV transmission networks and whether an adjustment in methods of genotype data collection can contribute to capturing the dynamic of the entire population even with low sampling.

## Methods

Here we studied how the gap in access to sequence data for all PWH influences the detection of clusters and priority clusters in molecular transmission networks, and consequently, lowers the effectiveness of public health interventions. We investigated how the structure of reconstructed networks changes with artificially lowered data completeness. We used two techniques of sampling for low data completeness: i) random subsampling without replacement and, ii) subsampling based on node influence (details in the next section). Additionally, we tested a method for extracting the patterns of transmission with artificially sub-sampled data for low completeness.

**Figure 1:**
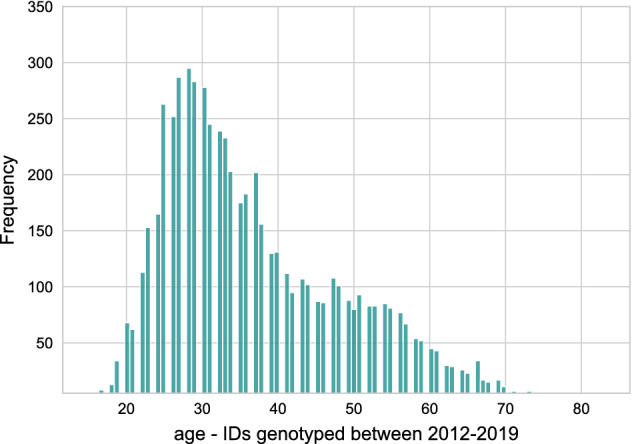
Metadata—age distribution of people with diagnosed HIV (2012–2019).

We analyzed HHD longitudinal surveillance data from 6852 PWH with pol sequences with an HIV diagnosis between 2012 and 2019. Each person resided in Houston, Kingwood (an annexed suburb), or Harris County at the time of HIV diagnosis or at some point after being diagnosed. People ≤ 13 years of age at the time of diagnosis or with a perinatal exposure risk were excluded. By comparing the ratio of the genotyped individuals to the overall number of PWH (12,818), we computed the data completeness to approximately 53.46%^15^. Table 1 and Fig. 1 demonstrate the distribution of their corresponding metadata. For cluster analysis of the molecular data, we removed the sequences shorter than 500 bp and used the TN93 substitution model to measure genetic distances using the first reported pol sequence per individual and reconstructed the molecular transmission network^22^. In this approach, each HIV molecular sequence is compared to every other HIV molecular sequence to identify pairs of sequences that are extremely similar (i.e., sequences that have a very small genetic distance, or difference). A total of 2257 individuals clustered at 1.5% substitutions per site. The majority of the detected clusters in the network were dyads and showed no growth over time. Hence, we measured the impacts of lower completeness on the detection of priority clusters, following the CDC’s standard approach in which people are only included in the analysis if diagnosed in the last three years and the genetic distance threshold equals 0.5% substitutions per site. Furthermore, to meet national priority criteria, clusters must possess ≥ 3 new HIV diagnoses in the past 12 months for lower burden jurisdictions (≥ 5 diagnoses for higher burden jurisdictions)^4,23^.

**Table 1:** Metadata—characteristics of the data and network of Houston/Harris County HIV surveillance data: 2012–2019.

	Count	%
**Gender**		
Cisgender men	5551	81
Cisgender women	1151	16.8
Transgender person (women, men)	150	2.2
**Race/Ethnicity**		
Black	3035	44.29
Hispanic, All races	2539	37.05
White	922	13.45
Multi-race	242	3.53
Asian	114	1.66
**Transmission category**		
Adult male-male sexual contact (MSM)	3947	57.6
No identified risk factor (NIR)	907	13.23
No risk factor reported (NRR)	893	13.03
Heterosexual contact	777	11.33
MSM and PWID	178	2.59
People Who Inject Drugs (PWID)	150	2.32
**Stage of the disease**		
HIV and later AIDS	2836	41.38
HIV only	2013	29.37
HIV and AIDS simultaneously	1932	28.19
Unknown	71	1.03

12,818 diagnosed people and 6852 unique sequences.

We calculated the average risk of infection per individual in priority clusters (using Eq. 1) in the full dataset and compared it with randomly subsampled data to test whether lower data completeness can influence our estimation of prospective infection rates.

1 $$ Risk_{inf} \left( {id} \right) = \frac{1}{m}\sum\nolimits_{i = 1}^{m} \frac{1}{n} \sum\nolimits_{t = 1}^{n} {\left( {\frac{{\left| {C_{it + 1} - C_{it} } \right|}}{{C_{it} }}} \right)} $$

where C_it_ denotes the size of cluster i in time t, for m clusters in n length of time.

**Figure 2:**
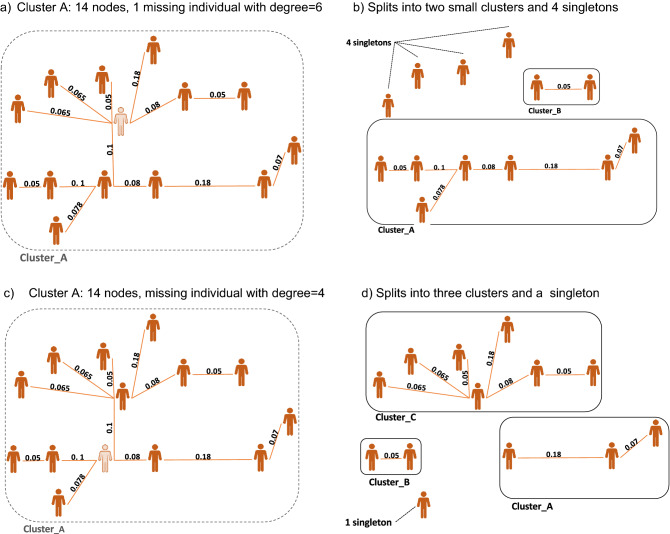
Example of how missing one node in a network can change its clustering dynamics: (**a**) cluster A with 14 individuals and their pairwise genetic distance, (**b**) missingness of one node (degree = 6), causes cluster A to split into two smaller clusters and also leaves 4 nodes as singletons, (**c**) repeating cluster A with another node missingness (degree = 4), (**d**) the new missingness, caused cluster A split into three smaller clusters and to leave one node as singleton.

Thus far, we investigated the impacts of random subsampling of the molecular data. The random sampling method assumes that all nodes have the same node influences in the transmission network, therefore only the presence of the sequence is accounted for when assessing probable impacts on the estimated transmission network. However, previous studies have shown that certain individuals contribute more to future transmission^24^. Consequently, random selection from full completeness might make the subsampled data analysis suffer from sampling bias. Figure 2 shows an example of two nodes with slightly different node influences in the network. Figure 2a shows one node (color-coded with light orange) which is part of one cluster consisting of 14 nodes. If the genotype of this node was not sampled, the network would split into two smaller clusters and 4 nodes become singletons (shown in Fig. 2b). Another example in the same network in Fig. 2c shows not having access to the genotype of another node (color-coded with light orange), would change the reconstructed network into 3 smaller clusters and leaves one node as a singleton (shown in Fig. 2d). Therefore, the missingness of only one node in the dataset can potentially change the structure of the reconstructed network in different ways, and simply counting how many nodes are collected or are missing in the dataset will not show the real impacts on the underlying transmission network. In the next section, we explain the details of our subsampling methods and the impacts of each technique are presented in the Results section.

### Subsampling method

First, we performed a random selection by removing records in tenfold without replacement (in 10% decrements) from the full dataset. The completeness of sequence coverage in our sub-sampled dataset ranges from 52% down to 15%. In every round of subsampling, we reconstructed the network again to compare its characteristics. In the second round of subsampling, we considered node influence to examine whether applying missingness of different individuals artificially has the same impact on the reconstructed network as all nodes having uniform spreading power in the network. To measure the node influence (or spreading power), we used a measure called Expected Force (ExF)^24^. ExF is an Eigenvector metric that measures the importance of a node based on the importance of its connections within the network. It computes the spreading power of individual nodes by adopting a relative influence of different walk and walk lengths based on local connectivity in a network. Therefore, the more critical connections a node possesses, the more critical the node becomes. We computed ExF for each node in the network ranging from 0 to 3. Then we removed the nodes with high ExF (≥ 1.9), which is slightly over the average ExF, and built the transmission network. Again, we repeated the process by removing the nodes with low ExF (≤ 1.5) to compare the resulting networks with the full dataset. We compared the detection of clusters in networks using the full dataset versus each subsampled method.

### Study setting and data availability

HIV molecular sequence data were reported from drug-resistance genotyping for people newly diagnosed with HIV while residing in Houston-Harris County. These samples were then stored in the Enhanced HIV/AIDS Reporting System. Reporting of HIV genotypic testing has been required by law (Tex. Adm. Code Chapter 97, Subchapter F, §97.133) since January 1, 2010. Data were collected through public health surveillance in accordance with relevant guidelines and state regulations of Texas administrative and health and safety code^25,26^. The experimental protocol was approved by the ethical committee in Houston Health Department^26^. The collection of these data falls under mandatory reporting guidelines for infectious diseases and does not require informed consent. This study was deemed to be IRB exempt (category 4) from institutional review by the Committee for the Protection of Human Subjects at the University of California San Diego because it was a retrospective analysis of surveillance data for the purposes of program evaluation. The de-identified data were analyzed in accordance with a Memorandum of Understanding for data sharing between the Houston Health Department and the University of California San Diego. All methods were carried out in accordance with relevant guidelines and regulations. The data that support the findings of this study are available from the Houston Health Department, but restrictions apply to the availability of these data. With the written permission of the Houston Health Department, data are available from the authors upon reasonable request. Permission from the Houston Health Department may be requested by contacting the Investigative Review Committee (analysisdatarequest@houstontx.gov). More: www.houstonhealth.org/about/investigative-review-committee.

## Results and conclusion

**Figure 3:**
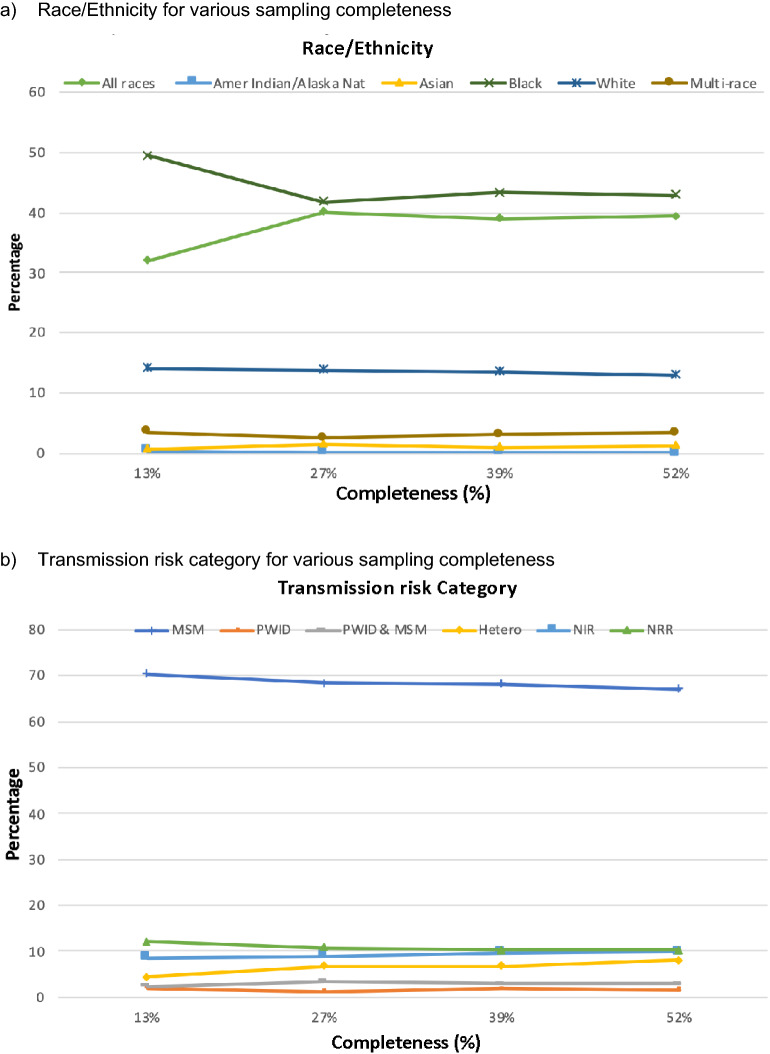

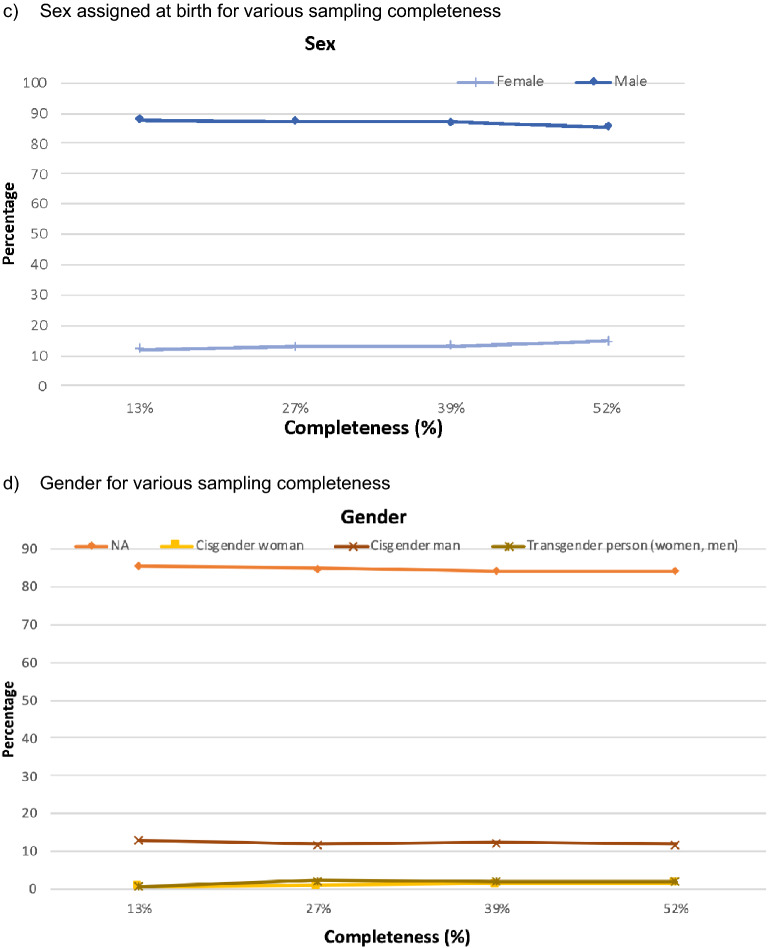
The distribution of clustered nodes in three demographic categories and transmission risk for full versus artificially subsampled data: (**a**) race/ethnicity, (**b**) transmission risk category, (**c**) sex (assigned at birth) and (**d**) gender.

Analyzing the sequencing data from 6852 individuals diagnosed between 2012 and 2019 and reported to the HHD, we detected 544 clusters ranging in size from 2 to 56 nodes. The genotype data completeness rate was estimated at 50% (computations explained in Sect. “Methods”). We compared the reconstructed network created from the full dataset against networks created with artificially reduced data. The distribution of subcategories in the race, transmission risk, sex assigned at birth, and gender remain relatively similar in the full dataset compared to randomly subsampled data (Fig. 3). These results indicate that the subsampling method did not substantially influence the distribution of race, transmission categories, sex assigned at birth, and gender in comparison to the full dataset.

**Figure 4:**
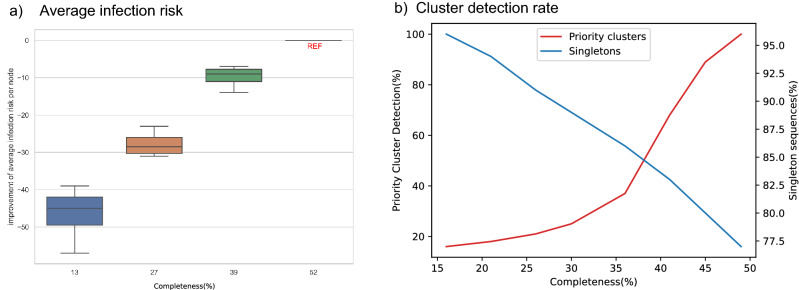
Houston/Harris County (2012–2019) data: (**a**) average infection risk improvement, (**b**) cluster detection trend (left y-axis) and Singleton sequence rate versus different data completeness rates (right y-axis) vs various genotype data completeness.

Priority clusters show recent and rapid transmission with a small genetic distance threshold (0.5% substitution per site) among people diagnosed in the most recent 3-year period. We compared the risk of infection per individual in priority clusters for full versus subsampled data. The results are shown in Fig. 4a. The estimated rate of infection risk for nearly 75% of subsampled data dropped by an average of 10% in comparison to full completeness, which is considered reasonable^27^. Also, it shows with data completeness of ≤ 50%, the average infection risk rate diminishes by 25–57%. These results show that not only the upper limit of completeness is important in seeking an accurate transmission network, but also that datasets ≤ 50% data completeness may not represent the underlying transmission network (Fig. 4b). With the manual random reduction of data completeness, the detection of clusters in general decreases in a linear trend (red line in Fig. 4b). Furthermore, we measured the rates of individuals being clustered or remaining as singletons with the alterations in the rate of completeness. The blue line in Fig. 4b shows with less than 30% completeness, the size of the reconstructed networks reduced drastically with over 85% of the individuals not clustering.

**Figure 5:**
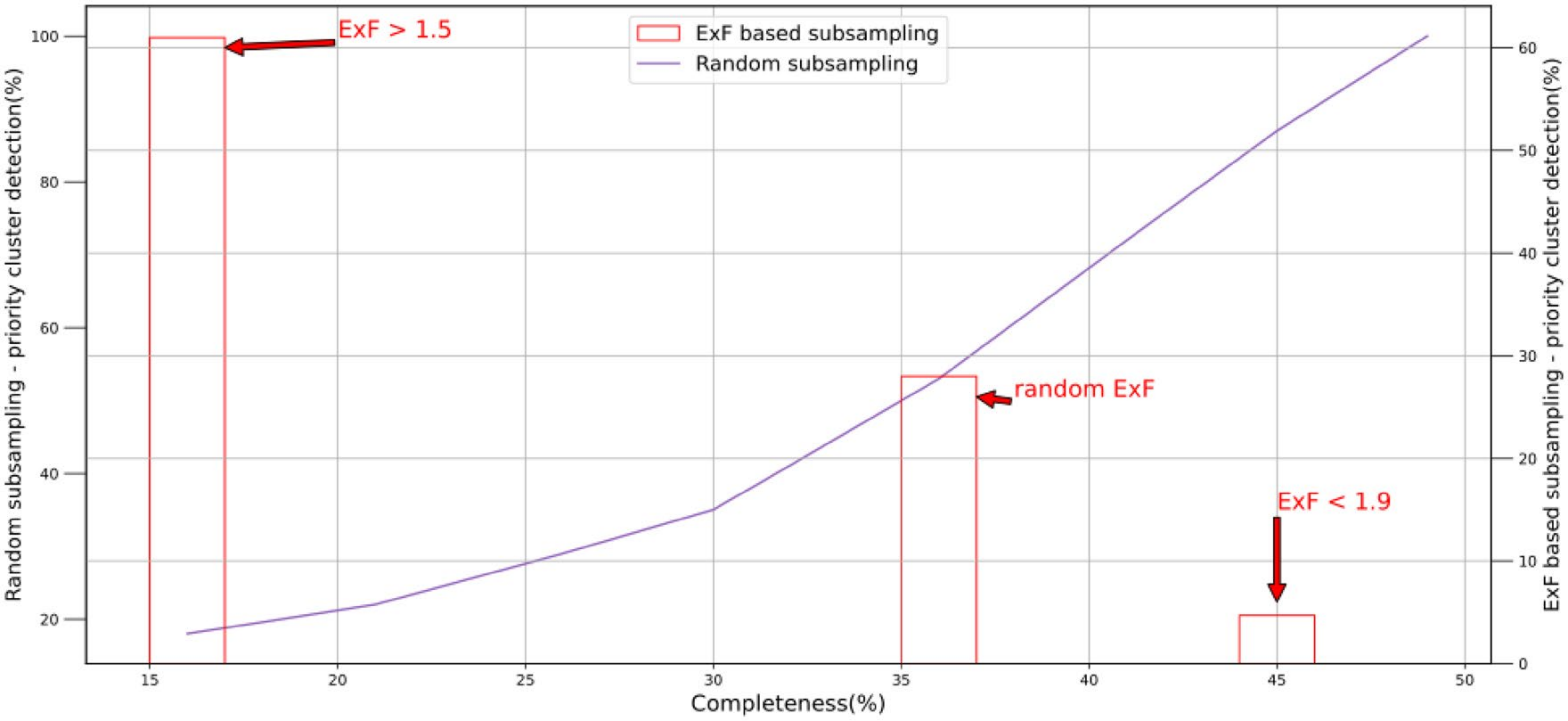
Cluster detection comparison for two sampling methods (Houston/Harris county 2012–2019): Left Y axis shows priority clusters detected in Randomly subsampled data, Right Y axis shows priority clusters detected from subsampled data based on Expected Force (ExF) node influence measure.

**Figure 6:**
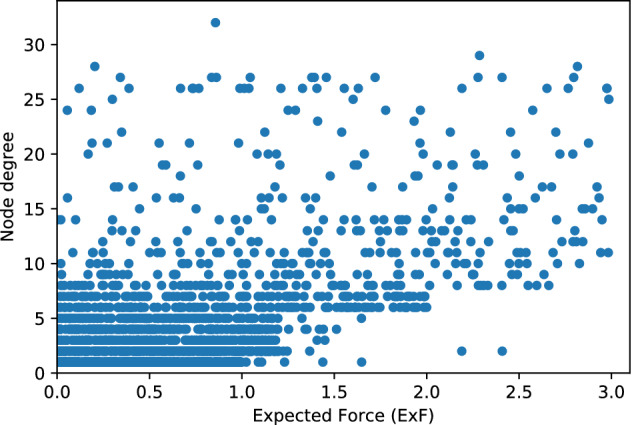
Node Influence (ExF) per Node degree distribution of clustered sequence—Houston Health Department: 2012–2019.

In order to test whether the gap of access to all genotypes in a molecular network will have the same impact on the accuracy of the reconstructed molecular transmission network, we computed the node influence (ExF) of 2257 clustered nodes in the network. Then we performed two rounds of subsampling. In the first round, we removed all nodes with an Expected Force of ≤ 1*.*5, reconstructed the network and determined the priority clusters detected in the network consisting of only high node influences. Our results showed that 61% of the priority clusters were detected with just 25% of sequences present in comparison with full data when we removed nodes with low influence. We repeated the analysis with the removal of nodes with ExF ≥ 1*.*9, which we considered high influential nodes and only 4.7% of the priority clusters were detected with approximately 75% of sequences in the dataset (shown in Fig. 5 in red ink). In the second round, we measured what percentage of priority clusters were detected using subsampling tenfold (shown in Fig. 5 in purple ink). Furthermore, we compared the degree for all clustered nodes in the network and plotted them versus the node influence. Figure 6 (created using synthetic data based on real data from HHD 2012–2019) shows that there is no clear pattern in correlation between node degree and the ExF of clustered nodes in our study. We plan to investigate for any probable non-linear relationship between them.

Despite global efforts in the collection of genotype data at diagnosis and utilizing it for retrospective analysis of HIV outbreaks, collection has not been possible due to limited funding and available resources in some parts of the world^28^. Evidently, having more data results in finding more clusters, but the tradeoff is finding a rate of genotype sampling in which a lower or higher level of completeness may not greatly improve the accuracy of the reconstruction method. Therefore, determining whether the data used for a study represents the dynamics of the entire underlying transmission network is an important consideration when strategically planning prevention interventions. It is essential to have reliable methods, as well as an adequate amount of data to make a fairly accurate estimation of retrospective transmissions, which will be used as the basis of many prospective analyses and HIV elimination efforts.

In any but the most homogeneously mixing populations, some individuals have a disproportionate impact on the size of an epidemic. This impact may, for example, be due to a high degree of connections (as in the case of highly connected nodes^29^), or due to having a critical role in joining up a network (as in a “bridge”)^30^. Node measures provide an imperfect, but useful, way of capturing some of these types of importance. The node influence method (ExF) has been effectively used in variety of epidemiological models and human interactive networks. The ExF is based on node degree in which low degree nodes influence depend on their neighbors degree, while high degree nodes are self-dependent. The strength of this relationship is modulated by network structure, being more pronounced in narrow, dense networks typical of social networking and weakening in broader, looser association networks such as the Internet^24^. The expected force can be computed independently for individual nodes, making it applicable for networks whose adjacency matrix is dynamic, not well specified, or overwhelmingly large^24^. For future research, more complex networks with non-linear dynamics should be explored by considering both local and global-level measures (small-world network topology)^30^ of potential impact of shortcuts that connect distinct clusters. The current study is the initial step to focus on local connectivity and its impact on influencing HIV transmission.

Here, we investigated whether low levels of data completeness in molecular genotype data can affect the accuracy of estimated transmission networks and whether it can influence the projection of future predictions. In this study, we performed multiple analyses to measure the network reconstruction method’s sensitivity in determining clusters of transmission with low completeness. Our results demonstrate that having a limited dataset can negatively impact HIV cluster detection with the current method of collecting and analyzing genotype data. These results are evidence that many key network features of a HIV transmission, such as the characteristic exponent of the scale-free distribution for linkage, can be reasonably estimated at low completeness (of ≤ 50% in this study) with a different methodology for inference.

Furthermore, having ≥ 75% of sequencing data can be considered a reliable representation of full data although not entirely equivalent. We showed that incompleteness in general limits our ability to capture highly connected nodes that impact the overall dynamic of the underlying transmission network. We hypothesize that an ideal range of completeness exists for cluster detection whereby less than the lower limit is not representative of the transmission network and higher than the upper limit of the range will not drastically improve cluster detection. Future analyses among areas with higher sequence completeness are recommended to investigate the possibility of capturing more of the highly connected nodes in their reconstructed transmission network compared to the jurisdictions with lower than 50% data completeness. This analysis could better confirm the incremental gains in cluster detection at higher completeness for public health responses. The implication of this study is to propose a network method that enables inference of the presence of “invisible” members of the transmission network that are not captured in real-world sampling. Such nodes could have a major impact on various network structures including the scale-free network 29 featured by a few hubs or highly connected nodes. Our research outputs are expected to inform effective network-based prevention and implementation strategies to eliminate HIV. Moreover, we extended this study to find an alternative method for identifying priority (recent and rapidly growing) clusters toward the goals of HIV elimination when access to molecular data is limited. Our results showed that a small but influential data set can still be effective to detect a majority of the priority clusters that show the dynamics of the underlying transmission network. This study lends credence to the notion that utilizing drug resistance data alone for detecting clusters may be similar to a random selection of data. Therefore, we suggest consideration of network metrics, such as node influence, in molecular cluster detection. There is a caveat though in using our proposed method, considering that individual’s attributes (e.g., racial/ethnicity minority group, younger age group) could be a potential driver for network connectivity in HIV transmission^31^. Future research merits considering such nodal attributes incorporated into the computation of influential nodes to assess their impact on cluster detection.

In brief, although maximum sequence completeness is ideal for cluster detection, this goal is hampered by delays in diagnosis, reporting completeness, and ordering practices by individual providers. The incompleteness of sequence data may also reflect access to care. Existing health services research or healthcare programs centered on increasing linkage and retention in care could be an avenue for partnership with public health departments whereby emphasis is placed on universal drug-resistance testing for both the individual and community-level benefit. While individuals may benefit from such testing for selection of treatment, community-level benefits may be realized through more complete data that better inform evaluations of varying methods of cluster detection, including those that incorporate node influence.
